# At the Edge of Chaos: How Cerebellar Granular Layer Network Dynamics Can Provide the Basis for Temporal Filters

**DOI:** 10.1371/journal.pcbi.1004515

**Published:** 2015-10-20

**Authors:** Christian Rössert, Paul Dean, John Porrill

**Affiliations:** Department of Psychology, The University of Sheffield, Sheffield, United Kingdom; Université Paris Descartes, Centre National de la Recherche Scientifique, FRANCE

## Abstract

Models of the cerebellar microcircuit often assume that input signals from the mossy-fibers are expanded and recoded to provide a foundation from which the Purkinje cells can synthesize output filters to implement specific input-signal transformations. Details of this process are however unclear. While previous work has shown that recurrent granule cell inhibition could in principle generate a wide variety of random outputs suitable for coding signal onsets, the more general application for temporally varying signals has yet to be demonstrated. Here we show for the first time that using a mechanism very similar to reservoir computing enables random neuronal networks in the granule cell layer to provide the necessary signal separation and extension from which Purkinje cells could construct basis filters of various time-constants. The main requirement for this is that the network operates in a state of criticality close to the edge of random chaotic behavior. We further show that the lack of recurrent excitation in the granular layer as commonly required in traditional reservoir networks can be circumvented by considering other inherent granular layer features such as inverted input signals or mGluR2 inhibition of Golgi cells. Other properties that facilitate filter construction are direct mossy fiber excitation of Golgi cells, variability of synaptic weights or input signals and output-feedback via the nucleocortical pathway. Our findings are well supported by previous experimental and theoretical work and will help to bridge the gap between system-level models and detailed models of the granular layer network.

## Author Summary

The cerebellum plays an important role in the learning of precise movements, and in humans holds 80% of all the neurons in the brain, due to numerous small cells called “granule cells” embedded in the granular layer. It is widely thought that the granular layer receives, transforms and delays input signals coming from many different senses like touch, vision and balance, and that these transformed signals then serve as a basis to generate responses that help to control the muscles of the body. But how the granular layer carries out this important transformation is still obscure. While current models can explain how the granular layer network could produce specific outputs for particular reflexes, there is at present no general understanding of how it could generate outputs that were computationally adequate for general movement control. With the help of a simulated granular layer network we show here that a random recurrent network can in principle generate the necessary signal transformation as long as it operates in a state close to chaotic behavior, also termed the “edge-of-chaos”.

## Introduction

Many models of the cerebellum assume that the granular layer recodes its mossy-fiber inputs into a more diverse set of granule-cell outputs [1–4]. It is further assumed that the recoded signals, which travel via granule-cell ascending axons and parallel fibers to Purkinje cells and molecular layer interneurons, are appropriately weighted using plastic synapses and then combined to produce the particular Purkinje cell outputs that are required for any given learning task. Recoding in these models thus enables a given set of mossy-fiber inputs to generate one of a very wide variety of Purkinje cell outputs, giving the model demonstrable computational power (e.g. [5]).

Although this framework is seen as plausible in broad outline (e.g. [6,7]), the details of its workings are far from established [8]. Relatively simple top-down models have shown that theoretically well-understood recoding schemes such as tapped delay lines, spectral timing, Gaussians, sinusoids, and exponentials can be effective, but do not establish how they could be implemented biologically (references in [8–10]). In contrast, more complex bottom-up models of recurrent inhibitory networks representing the connectivity between granule and Golgi cells are closer to biological plausibility, but have been used for very specific tasks such as eye-blink conditioning so that their general computational adequacy is unknown [11–20].

In part this is because eyeblink conditioning requires a response only at the time the unconditioned stimulus arrives. Eyelid (or nictitating membrane) position is not specified either for the period between the conditioned and unconditioned stimulus, or for the period (possibly some hundreds of milliseconds) after the unconditioned stimulus has been delivered. In contrast, for a task such as the vestibulo-ocular reflex eye-position is very precisely specified for as long as the head is moving, and afterwards for as long as gaze has to be held constant. Thus, cerebellar output—and hence granular-layer output—is more tightly constrained in motor-control tasks resembling the vestibulo-ocular reflex than in eyeblink conditioning [3].

Here we combine elements of top-down and bottom-up approaches, by investigating whether the outputs of neural networks that incorporate the recurrent inhibition observed in the granular layer can be linearly combined to generate continuous filter functions which are computationally useful for example in vestibulo-ocular reflex adaptation [9]. The split between a complex representation layer (granular layer) and a linear reconstruction layer (perhaps corresponding to the plastic synapses between granule cells and Purkinje cells or molecular-layer interneurons) is similar to the structure employed in reservoir computing [21], and it is convenient to use terminology and methods from that field in analyzing these networks (see Methods).

We begin by analyzing the case of a one-layer network with recurrent inhibition [15]. This is simpler than the real granular layer in which feedback is provided via a second layer of Golgi cell interneurons, but is worth analyzing separately because it allows us to test the hypothesis, suggested by the reservoir computing metaphor, that the crucial parameter in determining the time extension of responses is the mean amount of feedback in the network, and how closely this parameter is tuned to the edge-of-chaos [22]. This degree of tuning can be measured by the Lyapunov exponent. Generally speaking, if there is very little recurrent feedback in a network, then responses will be highly stable and die away very quickly over time, while for large amounts of feedback the responses can be chaotic or even unstable. The Lyapunov exponent (see Methods) is a quantitative measure of stability because it captures the rate of growth or decay of small perturbations. In linear systems negative values imply stability, while positive values imply instability. In non-linear systems, small, negative values of Lyapunov exponents can be especially interesting, since they can signal the ‘edge-of-chaos’, where there are long-lasting and possibly complex responses to transient inputs. We show that this is the interesting region for our reconstruction problem.

One novel feature of this contribution is its use of generic colored noise inputs, rather than the stereotyped pulse or step inputs that are usually considered. These colored-noise inputs are essential for motor control applications such as the VOR, where they are needed to demonstrate that the filter can process generic vestibular signals. A second novel feature is the use of statistical techniques that allow us to evaluate the ability of the network to approximate the range of linear filters required for these applications.

While previous work on reservoir networks focused on generic inhibitory and excitatory networks [22–30] this is the first work to systematically examine stability and reservoir performance in networks dominated by recurrent inhibition like the granular layer while also taking into account the effects of cerebellar network properties on filter approximations. To achieve this we extend the model to two populations in order to represent inhibition via Golgi cells. We also test the effect of other non-generic features of the cerebellum such as the newly discovered functional feature of Golgi cell inhibition by mGluR2 receptor activated GIRK channels [31,32] and Golgi cell afferent excitation often neglected in cerebellar simulations. Furthermore we also evaluate the effect of output-feedback to the granular layer through the nucleocortical pathway.

## Methods

### One population model

**Fig 1:**
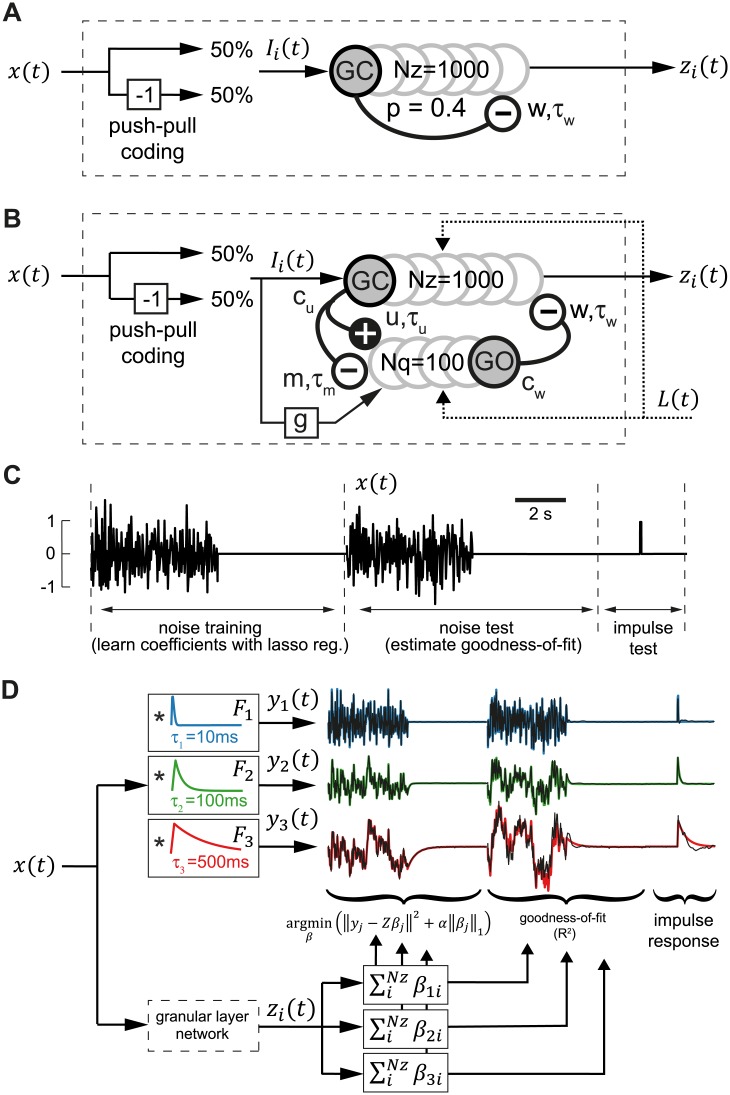
Granular layer models and filter construction procedure. **A:** Diagram of one-population granular layer model based on Yamazaki and Tanaka [15] consisting of mutually inhibiting granule cells and input signals coded by push-pull input. **B**: Diagram of two-population granular layer model consisting of granule cells (GC) and Golgi cells (GO). GC innervate GO using glutamatergic excitation (u) and inhibition by mGluR2 activated GIRK channels (m). GO inhibit GC by GABAergic inhibition (w). All synaptic connection simulated using single exponential processes (see Methods). **C**: Input signal *x*(*t*) consisting of colored noise input for training, test and impulse response input. **D**: Diagram of filter construction test procedure. The output signals *z*_*i*_(*t*) of all granule cells during the training sequence were used to construct exponential (leaky integrator) filters of increasing time constants *τ*_*j*_ = 10ms (blue line), 100ms (green line) and 500ms (red line) using LASSO regression (see Methods). The goodness-of-fit (*R* ^2^) of this filter construction was evaluated during the noise test sequence.

The one-population model used in this study (Fig 1A) was based on that of Yamazaki and Tanaka [15]. It consisted of *N*_*z*_ = 1000 granule cells, each receiving excitatory afferent inputs *I*_*i*_(*t*) derived from the external signal *x*(*t*), and recurrent inhibitory inputs from other cells. The model neurons were firing-rate (i.e. non-spiking), and the output *z*_*i*_(*t*) of the i-th neuron at time t was given by

$${\text{z}}_{\text{i}}\left(\text{t}\right)={\left[I_{i}\left(t\right)-\overset{\mathrm{Nz}}{\underset{j}{\sum^{}}}A_{\mathrm{ij}}w_{\mathrm{ij}}\overset{t}{\underset{s=1}{\sum^{}}}\text{exp}\left(-\frac{t-s}{\tau_{w}}\right)z_{j}\left(s-1\right)+{\mathrm{nN}}_{i}\left(t\right)\right]}^{+}$$ (1)

(here the bracket notation []^+^is used to set negative values to zero, preventing the firing-rate of a neuron from becoming negative). This equation describes (see Fig 1A) memory-less rate-neurons connected by single-exponential synaptic process with time constant *τ*_*w*_ so that neuron *i* sums past inputs *z*_*j*_(*s* − 1),1≤ s ≤ t from other neurons, exponentially weighted by distance *s* − *t* into the past. Neuron *j* has synaptic weight *A*_*ij*_ *w*_*ij*_ on neuron *i* where *A*_*ij*_ was set to 1 with probability *a* and 0 otherwise, hence the parameter *a* controls the sparsity of the connectivity. The connectivity strengths *w*_*ij*_ were drawn from a normal distribution with mean *w* and standard deviation *v*_*w*_ *w*, normalized by population size Nz, and constrained to be positive, so that $w_{ij}\;=\;{\left[\frac{2}{Nz}(w\pm v_{w}w)\right]}^{+}$. Each neuron received an excitatory input *I*_*i*_(*t*) with additive noise *nN*_*i*_(*t*) (here *N*_*i*_(*t*) is a discrete white noise process with *std*(*N*) = 1/2 so that the added noise is smaller in magnitude than the noise amplitude *n* 95% of the time).

In the simulations, unless otherwise specified, we used the following default values for the parameters above. The population size was *Nz* = 1000. The probability of connectivity was *a* = 0.4 (close to the value 0.5 in Yamazaki and Tanaka [15]), and synaptic variability was set to zero (*v*_*w*_ = 0). The default input noise level was *n* = 0.

This model had a single time constant which Yamazaki and Tanaka [15] took to be equal to the membrane time constant of Golgi cells in their simulations of granular layer dynamics. However it is not clear that this is the relevant time constant for a firing-rate model since the dynamics of the sub-threshold domain cannot be easily carried over into the supra-threshold (spiking) domain and are often counter intuitive. While a still prevailing misconception is that long membrane time-constants are equal to a slow spike response, the exact opposite is the case: integrate-and-fire with an infinite time constant (perfect integrators) have the fastest response time to a current step and can respond almost instantaneously [33]. Since temporal dynamics of neurons in a network are primarily determined by the time course of the synaptic currents [33–36] we have ignored membrane time constants in this and following models and instead related *τ*_*w*_ to the synaptic time constant of recurrent inhibition in the network.

We further want to note that the values for the synaptic time constants were not directly adjusted to replicate results for individual electrophysiological studies but rather kept at general values to study the effect on network output of interaction between different magnitudes of time constants. This issue is considered further in the Discussion.

### Two population model

To allow for more realistic modeling of the dynamics of the granular layer we extended the one-population network of granule cells above to include inhibition via a population of interneurons corresponding to Golgi cells (see Fig 1B). In this model the firing-rates *z*_*i*_(*t*) of granule cells and *q*_*i*_(*t*) of Golgi cells were given by

$$z_{i}\left(t\right)={\left[I_{i}\left(t\right)-\overset{Nq}{\underset{j}{\sum^{}}}A_{ij}w_{ij}\overset{t}{\underset{s=1}{\sum^{}}}\text{exp}\left(-\frac{t-s}{\tau_{w}}\right)q_{j}\left(s-1\right)+L\left(t\right)\right]}^{+}$$ (2)

$$q_{i}\left(t\right)={\left[g\cdot I_{i}\left(t\right)+\sum_{j}^{Nz}B_{ij}\left(u_{ij}\sum_{s=1}^{t}\text{exp}\left(-\frac{t-s}{\tau_{u}}\right)z_{j}\left(s-1\right)-m_{ij}\sum_{s=1}^{t}\text{exp}\left(-\frac{t-s}{\tau_{m}}\right)z_{j}\left(s-1\right)\right)+L\left(t\right)\right]}^{+}$$ (3)

The default sizes for the two populations were *Nz* = 1000 and *Nq* = 100. As before, the excitatory afferent input into a granule cell *i* was given by *I*_*i*_(*t*), however the two-population model also had direct afferent excitation *gI*_*i*_(*t*) of Golgi cells. The factor *g* setting the level of excitation was set to 0 in the initial simulations, resulting in no afferent excitation for Golgi cells. The output-feedback *L*(*t*) was 0 until later simulations (see below). The connectivity between the two populations was given by the random binary connection matrices *W* and *U*, however in this model the connectivity was not defined by a probability but by the convergence ratios *c*_*w*_ = 4 between Golgi and granule cells and *c*_*u*_ = 100 vice versa. Thus exactly 4 randomly selected Golgi cells inhibited each granule cell and 100 randomly chosen granule cells were connected to each Golgi cell. The weight of GABAergic inhibition between Golgi and granule cells was drawn from a normal distribution and normalized with ${w_{ij}\;=\;\left[\frac{2}{c_{w}}(w\pm v_{w}w)\right]}^{+}$(default *v*_*w*_ = 0) and the time constant of inhibition was given by *τ*_*w*_. Besides the glutamatergic excitatory connections between granule and Golgi cells with weight ${u_{ij}\;=\;\left[\frac{2}{c_{u}}(u\pm v_{u}u)\right]}^{+}$ (default *v*_*u*_ = 0) and time constant *τ*_*u*_ the model was extended to emulate the inhibitory effect of mGluR2 activated GIRK channels [31] with ${m_{ij}\;=\;\left[\frac{2}{c_{b}}(m\pm v_{m}m)\right]}^{+}$ (default *m* = 0, *v*_*m*_ = 0) and time constant *τ*_*m*_ = 50ms. Note that mGluR2 inhibition was not used until later simulations with *m* = 0.003. Additional simulations were conducted with only half of the Golgi cells receiving mGluR2 inhibition i.e. *Pr*(*m* = 0) = 0.5.

In all simulations *u* was set to 0.1 and normalized by the excitatory time constant resulting in *u* = 0.1/*τ*_*u*_.

All network simulations were written in C and were integrated into Python by transforming them into dynamically linked extensions with the package *distutils*. The stepsize in all simulations was *dt* = 1*ms*. All results were analyzed using Python. All models, methods and simulation results are available from the github repository https://github.com/croessert/ClosedLoopRoessertEtAl. A snapshot of the model code can also be found on ModelDB: https://senselab.med.yale.edu/modeldb/ShowModel.asp?model=168950. Computational resources for the simulations were partially provided by the ICEBERG cluster (University of Sheffield; access granted by the INSIGNEO Institute for in silico Medicine).

### Input

The modulated input to each cell was given by the excitatory input *I*_*i*_(*t*) = [*I*_0i_ + *f* ∙ 0.1 ∙ *I*_0i_ ∙ *x*(*t*)]^+^. Unless noted otherwise the input *I*_0i_ was chosen from a normal distribution with mean 1 and default standard deviation *v*_*I*_ = 0.1. To test increased input variability, standard deviation was increased to *v*_*I*_ = 2 in a later experiment. The factor *f*, randomly picked as either 1 or -1 defined whether the input was inverted or not. This type of input coding, here termed “push-pull” coding can be routinely found for example in the vestibulo-cerebellum where half of the cells are ipsilateral preferring (*f* = 1, type I) or contralateral preferring (*f* = −1, type II) [37].

In order to test the ability of the network to construct a linear filter with a given impulse response it is not sufficient to use impulse inputs alone, since this does not test linearity (for example the response to two successive impulse inputs may not be the sum of the individual responses). For this reason we also used random process inputs that mimic behavioral inputs.

The input signal *x*(*t*) consisted of 3 parts (see Fig 1C). The first part was a training sequence of a 5 second band-passed white noise signal (low-passed with a maximum frequency of 20 Hz) [38] chosen to mimic head velocity in the behaviorally relevant frequency range of 0–20 Hz [39]. Additionally a 5 second silent signal (*x*(*t*) = 0) was added to the training sequence to train a stable response. Training with a segment of null data finds weights which not only give the appropriate impulse response but also produce zero output for zero input data, so that they reject spontaneous modulatory activity in the network. Consecutively the previous signals were repeated with a different realization of the noise signal to test the quality of the filter construction. The third part was an impulse test signal where *x*(*t*) = 0 apart from a brief pulse of 50 ms where *x*(*t*) = 1. The colored noise signal was normalized to *std*(*x*) = 1/2 which ensured that the amplitude 0.1 ∙ *I*_0i_ included the input 95% of the time.

### Filter construction and quality measure

To assess the ability of the network to implement linear filters that depend on the past history of the inputs, the output signals *z*_*i*_(*t*) of all granule cells during the training sequence were used to construct exponential (leaky integrator) filters *y*(*t*) = *F* * *x*(*t*) of increasing time constants as linear sums *y*(*t*) = ∑*β*_*i*_ *z*_*i*_(*t*) of granule cell outputs. This can be regarded as the output of an artificial Purkinje cell that acts as a linear neuron. In matrix terms (writing time series in columns) this expression can be written *y* −*Zβ* where the undetermined coefficients *β* are usually fitted by the method of least squares to minimize root sum square fitting error

$$∥y-Z\beta {∥}_{2}=\sqrt{\underset{}{{\sum_{t}{\left(y\left(t\right)-{\Sigma^{}}^{}\beta_{i}z_{i}\left(t\right)\right)}^{2}}^{}}}$$ (4)

However over-fitting of the data, due to the large output population, can make this method misleading and give excessively high estimates of reconstruction accuracy. To avoid this problem we used the method of LASSO regression taken from the reservoir computing literature. This is a robust fitting procedure that includes a regularization term to keep the reconstruction weights small [40,41]. Here, the estimates are defined by $\hat{\beta }\;=\;{\text{argmin}}_{\beta }\left({\left\|y-Z\beta \right\|}^{2}+\alpha {\left\|\beta \right\|}_{1}\right)\;$ which is the least-squares minimization above with the additional constraint that the *L* ^1^-norm ||*β*||_1_ = ∑*β*_*i*_ of the parameter vector is also kept small. In practice we find that up to about 90% of weights are effectively zero using this method. In contrast to ridge regression that employs a *L* ^2^ -norm penalty and is commonly used to prevent over-fitting in reservoir computing [27] LASSO regression produces very sparse weight distributions. This corresponds well to the actual learning properties of the Purkinje cell, approximated as a linear neuron, in which optimality properties of the learning rule with respect to input noise force the majority of synapses to silence [42–45].

We fitted three responses *y*_*j*_(*t*) = *x*(*t*)* *F*_*j*_(*t*) with *j* = 1,2,3 and with *F*_*j*_(*t*) = exp(−*t*/*τ*_*j*_) being one of three exponential filters *τ*_*1*_ = 10*ms*, *τ*_*2*_ = 100*ms* or *τ*_*3*_ = 500*ms* (see Fig 1D). The regularization coefficient was set to *α* = 1*e* ^−4^ which gave best maximum mean goodness-of-fit results for the one-population model with *τ*_*w*_ = 50*ms* (not shown). LASSO regression was implemented using the function *sklearn*.*linear_model*.*Lasso()* from the python package *scikit-learn* [46].

In general the estimated weights *β*_*i*_ take both positive and negative values, which is not compatible with the interpretation of equation (4) above as parallel fiber synthesis by Purkinje cells. The use of negative weights is usually justified by assuming a relay through inhibitory molecular interneurons [42,44]. To test whether learning at parallel fibers alone is sufficient for the construction of filters from reservoir signals we additionally employed LASSO regression with only positive coefficients (positive-LASSO) as a comparison.

As a measure of the quality of filter construction, the weights estimated from the training sequence were used to construct the filtered responses in the test sequence and the goodness-of-fit between expected output and constructed output was computed for each filter using the squared Pearson correlation coefficient (*R* ^2^) [47] (see Fig 1D). For the final goodness-of-fit measure the mean of 10 networks with identical properties but with different random connections was computed.

### Lyapunov exponent estimation

A convenient way to analyze the stability or chaoticity of a dynamic system is the Lyapunov exponent *λ*. It is a measure for the exponential deviation of a system resulting from a small disturbance [25] and a value larger than 0 indicates a chaotic system. The Lyapunov exponent was measured empirically, similar to Legenstein and Maass [22] by calculating the average Euclidian distance $d\left(t\right)\;=\;\sqrt{\sum_{i\;=\;1}^{Nz}{\left(z_{i}\left(t\right)-z_{i}^{'}(t)\right)}^{2}}$ between all granule cell rates *z*_*i*_(*t*) from a simulation where *x*(*t*) = 0 and the rates $z_{i}^{'}(t)$ from a second simulation where the input was disturbed by a small amount at one time step, i.e. *x*(0) = 10^−14^. This state separation simulation was repeated for 10 randomly connected networks but otherwise identical parameters and *λ* was estimated from the mean average Euclidian distance $\overset{-}{d}\left(t\right)$ with $\lambda \;=\;{\text{log}}_{2}\left(\text{mean}(\overset{-}{d}\left(t\;=\;2.01s:2.11s\right))/\text{mean}\left(\overset{-}{d}\left(t\;=\;0.01s:0.11s\right)\right)\right)/2s$. To estimate the transition between stability and chaos we were mainly interested in the sign of the Lyapunov exponent. Although taking the mean of a 100 ms period and using a relatively large Δ*t* of 2s [24] decreases the accuracy of the Lyapunov estimation, it was used here to prevent errors in the estimation of the sign. The edge-of-chaos was defined as the point where *λ* crosses 0 for the first time when traversing in the direction of strong inhibition *w* to weak and therefore from high *λ* to low.

### Output feedback

To model putative output-feedback to the reservoir via the nucleocortical pathway the signal *L*(*t*) = *f* ∙ *o*_*i*_ ∙ −∑*β*_*i*_ *z*_*i*_(*t*) was injected into 20% of all granule and Golgi cells in the last simulations. The factor *f* was randomly picked as either 1 or -1 to model 50% excitation and inhibition and the weight was drawn from a normal distribution with *o*_*i*_ = [1*e* ^−4^±1*e* ^−5^]^+^. In these simulations only the case for output-feedback of the slowest filter signal is shown. Thus *β*_*i*_ are the weights needed to construct the filter with *τ*_3_ = 500*ms*.

As noted in the reservoir computing literature [27,48,49] output-feedback in general is a very difficult task since it leads to instability. Therefore the weights *β*_*i*_ were not learned online but a method called teacher forcing with noise was applied [27]. The weights *β*_*i*_ were learned in a prior step by using the teacher signal *L*′(*t*) = *f* ∙ *o*_*i*_ ∙ −*y*_3_(*t*) ∙ *N*(*t*) instead of the feedback signal *L*(*t*) to uncouple the instable learning. Here *y*_3_(*t*) is the target response for the slowest filter (Fig 1D) and *N*(*t*) is a discrete white noise process that helps to increase the dynamical stability [27]. The quality of filter construction and the Lyapunov exponent were estimated in a second simulation using the previously learned weights *β*_*i*_ for filter construction and the feedback signal *L*(*t*).

## Results

This section describes how filters with different time constants can be constructed from the activity of granular-layer-like networks of randomly connected neurons with recurrent inhibition, first for the one-population model consisting solely of simulated granule cells, then for the two-population model with both granule and Golgi cells.

### One-population model

In the first part of this study we focused on the one-population rate-neuron model previously published by Yamazaki and Tanaka [15]. While in this previous study the model was used to represent the passage of time, i.e. an internal clock, we now show that it is also possible to use its output to construct exponential filters with various time-constants.

**Fig 2:**
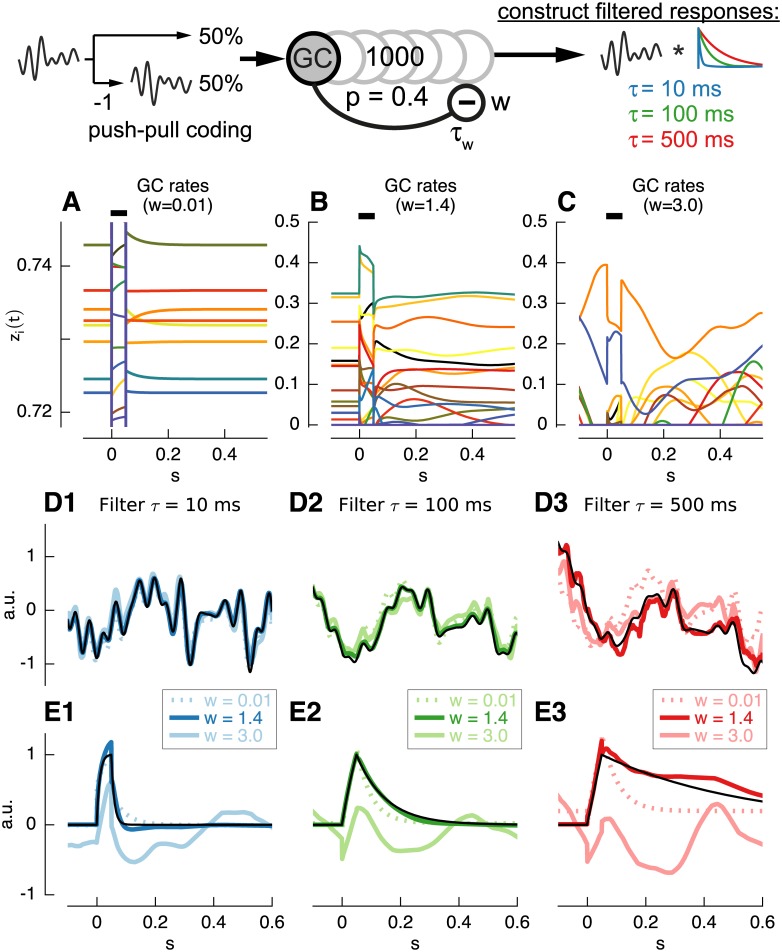
Responses of constructed filters and individual granule cell rates. **A,B,C**: Individual responses of randomly selected granule cells with weights *w* = 0.01 (**A**), *w* = 1.4 (**B**), *w* = 3 (**C**). Black bars indicate duration of pulse input. **D**,**E**: Responses of filters (*τ*_1_ = 10*ms* (**D1**,**E1**), *τ*_2_ = 100*ms* (**D2**,**E2**) and *τ*_3_ = 500*ms* (**D3**,**E3**)) constructed from network with inhibitory time constant *τ*_*w*_ = 50*ms* and inhibitory weight *w* = 1.4 (blue, green and red lines), *w* = 3 (light blue, light green and light red lines) or *w* = 0.01 (dotted light blue, light green and light red lines) to colored noise input (**D**) or pulse input (**E**). Responses of corresponding ideal filters shown as black lines. To construct the shown filters of 10/100/500ms the percentage of weights equal 0 and mean absolute weights > 0 was 90/53/34% and 20.6/60.4/86.1 for *w* = 0.01, 76/70/65% and 4.2/13.6/26.5 for *w* = 3 and 91/86/75% and 5.5/11.7/52.6 for *w* = 1.4, respectively.

To illustrate the dependence of network stability regime on the amount of feedback we begin by presenting sample impulse responses (Fig 2, second row) for a network (Fig 2, top) with intermediate time constant *τ*_*w*_ = 50*ms* and with three values of the recurrent inhibition: *w* = 0.01, lying in the highly stable region, *w* = 1.4, close to the edge-of-chaos, and *w* = 3, in the chaotic region. When the weight *w* was low, (Fig 2A, *w* = 0.01) the network was highly stable to perturbations and showed no long lasting responses. Close to the edge-of-chaos (Fig 2B, *w* = 1.4) complex, long lasting responses were present. For larger weights (Fig 2C, *w* = 3) the network entered a chaotic state in which cells showed random activity without further input modulation.

We further illustrate this dependence in the last two rows of Fig 2 which shows filter constructions (see Methods) for three target exponential filters with time constants *τ*_*i*_ of 10 ms (Fig 2D1 and 2E1), 100 ms (Fig 2D2 and 2E2) and 500 ms (Fig 2D3 and 2E3) (chosen to cover the range of performance required for e.g. VOR plant compensation [9]; filter construction of intermediate time constants are not shown, but are generally of similar quality). It is clear that in the highly stable regime only fast and intermediate time constant responses could be reconstructed (dotted light lines). Near the edge-of-chaos acceptable reconstructions were possible at all three time constants (dark lines), and in the chaotic regime reconstruction was always inaccurate and showed oscillatory artifacts (solid light lines).

While Yamazaki and Tanaka [15] argued that this chaotic network state is the preferred network state to implement an internal clock (compare Fig 2C with Fig 1 from [15]) these results show that it is disadvantageous when a filter of a continuous signal has to be implemented (see Discussion).

We have noted above (Methods) that accurate reconstruction of the impulse response of a linear filter does not imply that the output for other inputs is correct; this requires linearity of the reconstructed filter. Linearity of the reconstructed filters is investigated in the second row of Fig 2 by comparing their effects on a band-passed noise signal with that of the exact filter (plotted in black), again for time constants *τ*_*j*_ of 10 ms (Fig 2D1), 100 ms (Fig 2D2) and 500 ms (Fig 2D3), It is clear that the reconstruction in the stable regime or the chaotic regime (light lines) were much less accurate than in the edge-of-chaos-regime (dark lines). Note these plots show the response to a test input (rather than the training input, see Methods).

The regularized fitting method used (LASSO regression, see Methods) tends to use weights that are as small as possible. This property is clear in our example, to construct filters from granule cell signals at the edge-of-chaos only a small subset of granule cell responses were necessary. For the filters with 10, 100 and 500 ms (Fig 2D and 2E; *w* = 1.4), the percentage of weights being equal to zero was 90%, 86% and 75%, respectively, and the mean of non-zero weights was 5.5 and 11.7 and 52.6, respectively. The high proportion of silent synapses is consistent with experimental findings (see Discussion)

**Fig 3:**
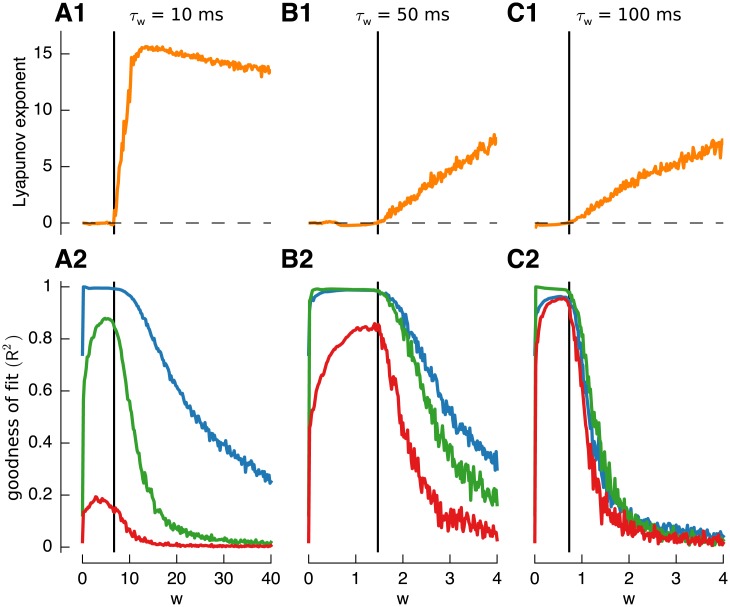
Construction of basis filters using a randomly connected network with feed-forward inhibition using LASSO regression. **A1,B1,C1**: Lyapunov exponent of randomly connected networks with increasing weight *w*. Networks with three different feed-forward inhibition time-constants are considered: *τ*_*w*_ = 10*ms* (**A1**), *τ*_*w*_ = 50*ms* (**A2**) and *τ*_*w*_ = 100*ms* (A3). Vertical black lines visualize the “edge-of-chaos” as defined in Methods. **A2,B2,C2**: Goodness of fit (*R* ^2^) of three exponential filters (*τ*_1_ = 10*ms* (blue), *τ*_2_ = 100*ms* (green) and *τ*_3_ = 500*ms* (red)) constructed from responses of corresponding networks above.

As discussed previously, the value of *w* corresponding to the edge-of-chaos can be identified using the Lyapunov exponent (see Methods). We illustrate this property by investigating the dependence of filter reconstruction accuracy on the Lyapunov exponent (Fig 3). Results are shown for three networks with different time constants for the recurrent inhibition: *τ*_*w*_ = 10*ms* (column 1), *τ*_*w*_ = 50*ms* (column 2) Fig 2B and *τ*_*w*_ = 100*ms* (column 3) approximately corresponding to the ranges of membrane and synaptic time constants present in the granular layer.

The top row of Fig 3 shows the Lyapunov exponent of each network plotted against the amount of recurrent inhibition *w*. In each case there was a point at which the exponent crossed the zero axis, corresponding to the edge-of-chaos value for that network time constant. It can be seen that the amount of recurrent inhibition needed decreased as the time constant increased.

The bottom row shows the effect of *w* on reconstruction accuracy (measured by *R* ^2^ goodness-of-fit) for exponential filters with the three time constants considered previously: *τ*_*j*_ = 10ms (blue lines), 100*ms* (green lines) and 500*ms* (red lines) for each network. Performance strongly depended on the weight of the recurrent inhibition. The goodness-of-fit was best, especially for filters with time constants longer than the internal inhibitory time-constant, for networks close to the edge-of-chaos, just before the transition from stable to chaotic behavior.

Other observations were that while, as expected, the goodness-of-fit for slow filters, e.g. 500*ms*, increased with the (inhibitory) time constant, the performance for fast filters decreased slightly (Fig 3C2). Furthermore the performance was best if the inhibitory time constant was equal to the time-constant of the filter (Fig 3A2, *τ*_1_ = 10*ms* blue line; Fig 3C2, *τ*_2_ = 100*ms* green line).

**Fig 4:**
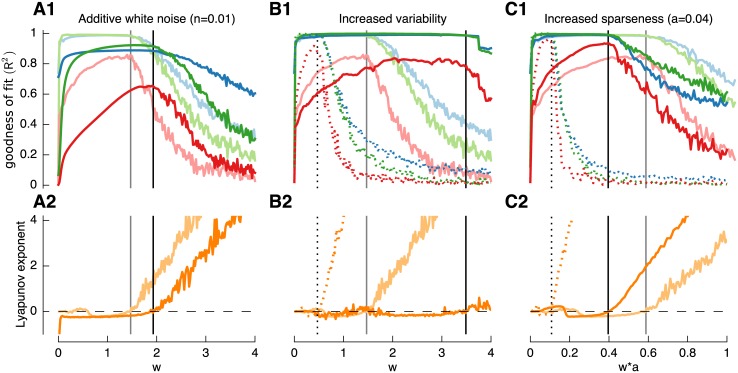
Filter construction is sensitive to various parameters. As previously, filters used are *τ*_1_ = 10*ms* (blue), *τ*_2_ = 100*ms* (green) and *τ*_3_ = 500*ms* (red). Default inhibitory time constant: *τ*_*w*_ = 50*ms*. Goodness of fit (*R* ^2^) (**A1,B1,C1**) and Lyapunov exponent (**A2,B2,C2**) are compared to a control shown as light solid lines in all subplots. **A**: Effect of additive white noise (n = 0.01) in the network (dark lines). **B**: Effect of increased input variability (v_*in*_ = 2) (dark solid lines) and increased variability of the inhibitory weight (v_*w*_ = 2) (dotted lines). **C**: Effect of increased sparseness by reducing probability of connectivity to *a* = 0.04 for network size *N* = 1*k* (dotted lines) and *N* = 10*k* (dark solid lines). For a better comparison x-axis was normalized with the probability of connectivity a.

Fig 4 investigates the robustness of the properties described above to moderate levels of additive noise and to variability in input signal levels and synaptic weights. While white noise with amplitude of *a* = 0.01 (noise amplitude equal to 10% of the input modulation amplitude) lead to a reduction in goodness-of-fit (Fig 4A1) the principal mechanism of filter construction was not disrupted and the edge-of-chaos was only shifted to larger weights *w* (Fig 4A2). Increasing the between-neuron variability of the mean input excitation to a high value of e.g. *v*_*I*_ = 2 (i.e. 95% of constant input increased to 0–5 from 0.8–1.2 for default value *v*_*I*_ = 0.1) (Fig 4B1 solid dark lines) had almost no benefit for the goodness-of-fit while shifting the edge-of-chaos to larger weight values.

In contrast, imposing larger variability in the inhibitory weight with *v*_*w*_ = 2 (i.e. 95% of weights between 0 and *w+*4*w*) shifted the edge-of-chaos in the opposite direction—towards lower weights (Fig 4B2, dotted lines), and the quality of filter construction was increased (Fig 4B1, dotted lines). This phenomena may be caused by a proportion of input signals or weights being driven to zero due to the positive cut-off which effectively leads to some cells receiving no input and a reduction of connectivity, respectively. To test the effect of reduced connectivity we examined the direct effect of increased sparseness on reservoir performance (Fig 4C).

Two methods were used to increase sparseness: the first was to decrease the convergence of inhibition to 40 cells (Fig 4C1, dotted lines) by decreasing the network connectivity from *a* = 0.4 to *a* = 0.04 while keeping the network size at *Nz* = 1*k*. The second way was to increase the network size to *Nz* = 10*k* while keeping convergence constant at 400 cells (Fig 4C2, solid dark lines) with *a* = 0.04. While both cases resulted in an improvement of filter quality, a smaller convergence slightly outperformed an increased network size suggesting that a sampling from less cells is more beneficial since it leads to a higher diversity and variability.

**Fig 5:**
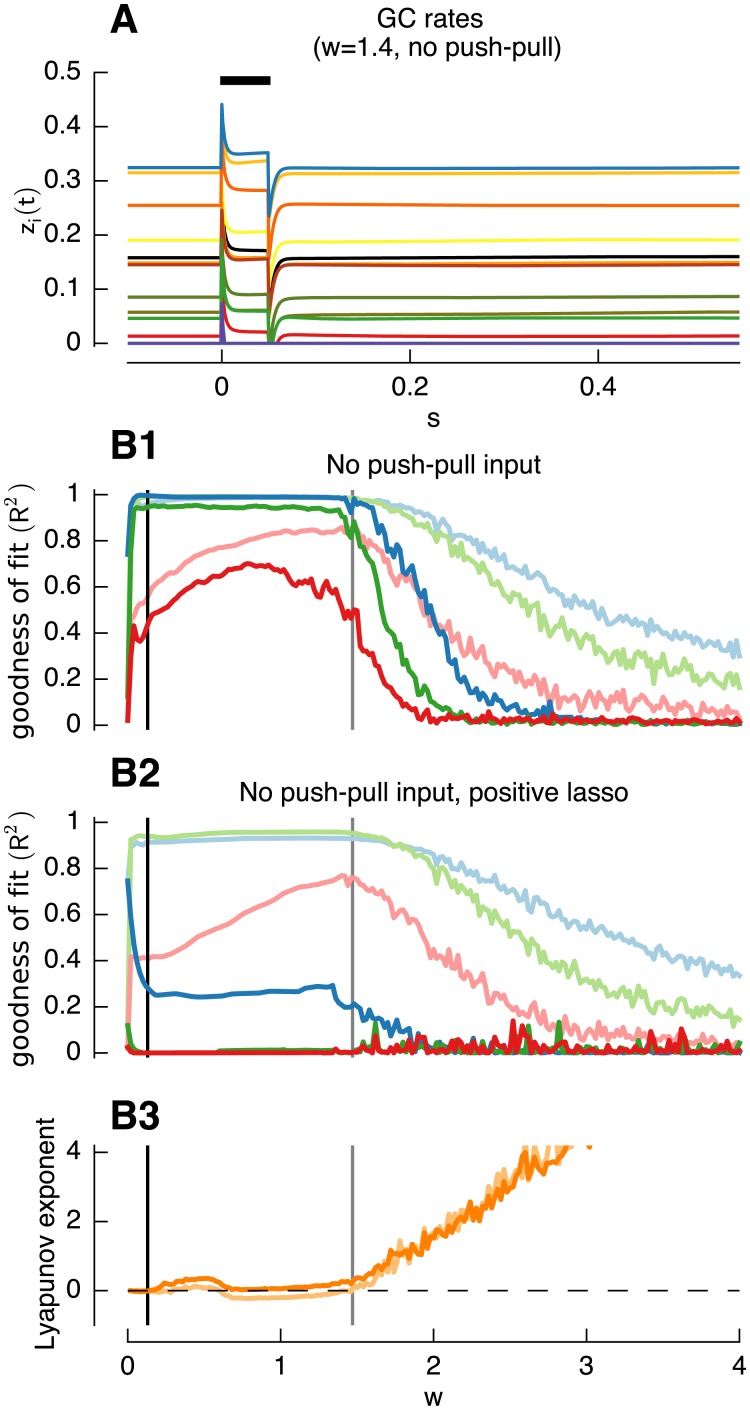
Push-pull coding is beneficial for filter construction. **A**: Individual responses of randomly chosen granule cells with *w* = 1.4 but without push-pull input coding. Black bars indicate duration of pulse input. **B**: As previously, filters used are *τ*_1_ = 10*ms* (blue), *τ*_2_ = 100*ms* (green) and *τ*_3_ = 500*ms* (red). Default network: *τ*_*w*_ = 50*ms* and push-pull input coding. **B1**: Default goodness of fit (*R* ^2^) (light lines) is compared to network without push-pull input coding (dark lines). **B2**: Goodness of fit for default network (light lines) is compared to network without push-pull input (dark lines) using regression with positive coefficients only. **B3**: Corresponding Lyapunov exponent for network with (dark orange line) and without push-pull coding (light orange lines).

An important requirement for filter construction turned out to be push-pull coding, found for example in the vestibulo-cerebellum, where half of the input signals are inverted (see Discussion). When the input did not include inverted signals the responses from individual granule cells showed almost no variety in damped oscillations in response to pulse input (Fig 5A). This consequently lead to an impairment of filter construction performance especially for larger filter time-constants and a shift of the edge-of-chaos to lower weights *w* (Fig 5B1, dark lines) when compared to the control case (light lines). Although filter construction performance was only slightly reduced when using regression with positive coefficients only (see Methods) (Fig 5C, light lines) when push-pull input was present, without push-pull input filter construction quality was heavily reduced (Fig 5C, dark lines).

### Two population model

While the previous model was able to show the principles of filter construction from a simplified model of the granular layer with recurrent inhibition, it did not take into account the fact that inhibition in the granular layer is relayed via a second population of cells, i.e. Golgi cells. To investigate the effects of this arrangement we extended the one-population model to a two-population model.

**Fig 6:**
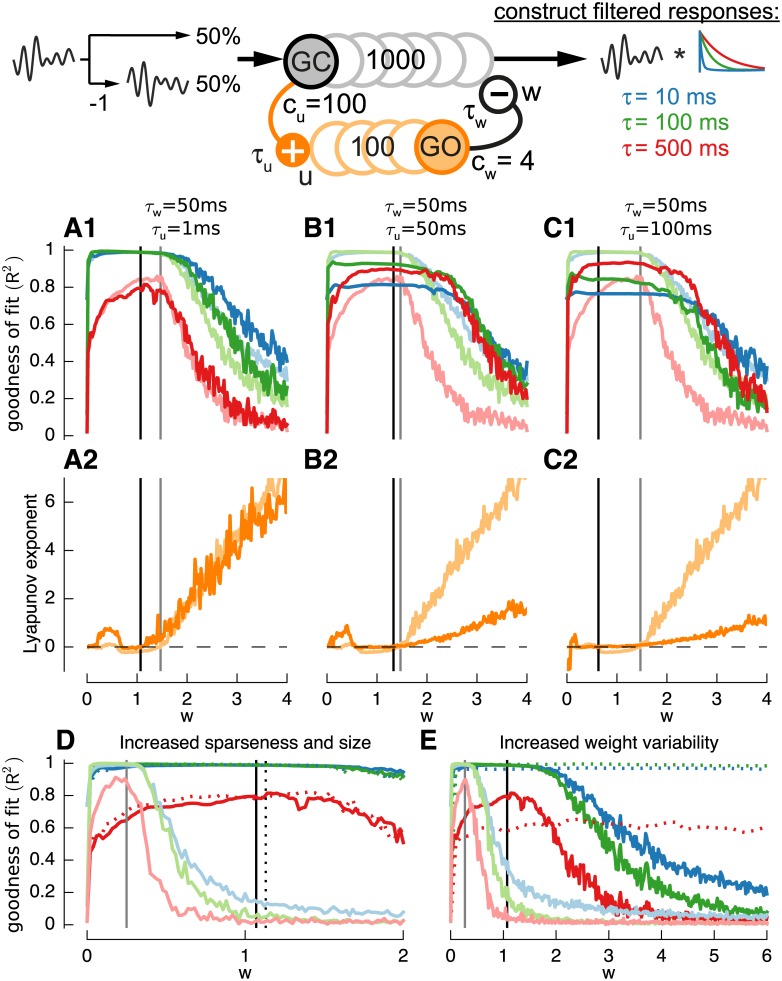
Construction of basis filters using a randomly connected network with feed-forward inhibition via a second population mimicking Golgi cells. As previously, filters used are *τ*_1_ = 10*ms* (blue), *τ*_2_ = 100*ms* (green) and *τ*_3_ = 500*ms* (red). **A,B,C**: Goodness of fit (*R* ^2^) (**A1,B1,C1**) and Lyapunov exponents (**A2,B2,C2**) for three networks with *τ*_*w*_ = 50*ms*, *τ*_*u*_ = 1*ms* (**A**), *τ*_*w*_ = 50*ms*, *τ*_*u*_ = 50*ms* (**B**) and *τ*_*w*_ = 50*ms*, *τ*_*u*_ = 100*ms* (**C**). Results for previous one-population network with *τ*_*w*_ = 50*ms* shown as light lines. **D**: Effect of increased sparseness in a two-population network with *τ*_*w*_ = 50*ms*, *τ*_*u*_ = 1*ms*: 1. Increase of granule cell population size from *Nz* = 1*k* (dark solid lines) to *N* = 10*k* (dotted lines). 2. Decrease of convergence to *c*_*u*_ = 10 while keeping network size at *Nz* = 1*k* (light solid lines). **E**: Effect of increased weight variability in a two-population network with *τ*_*w*_ = 50*ms*, *τ*_*u*_ = 1*ms*. Compared to control network without weight variability (dark solid lines). 1. Increase of variability for weight of inhibition w (*v*_*w*_ = 4) (dotted lines). 2. Increase of variability for weight of excitation u (*v*_*u*_ = 4) (light lines)

The connectivity of the extended model was based on plausible convergence ratios of *c*_*w*_ = 4 between Golgi and granule cells and *c*_*u*_ = 100 vice versa [50]. Additional parameters were excitatory time constant *τ*_*u*_ and the weight of excitation *u* (Fig 6, top). Increasing *τ*_*u*_ while keeping the inhibitory time constant at *τ*_*w*_ = 50*ms* showed that the performance of the two-population model was very similar to the one-population model if the excitation is fast (Fig 6A1). However, increasing the excitatory time constant improved the quality of the constructed slow filter (*τ* = 500*ms*) at the expense of the faster filters (*τ* = 10*ms* and *τ* = 100*ms*) (Fig 6B1 and 6C1). Additionally, this leads to a lowered gradient of the Lyapunov exponent (Fig 6B2 and 6C2). We therefore focus in the following on the best-case scenario of *τ*_*u*_ = 1*ms* and *τ*_*w*_ = 50*ms*. As in the one-population model before (Fig 2B), responses of single granule and Golgi cells in networks close to the edge-of-chaos featured complex but stable, long lasting damped oscillations (not shown).

In Fig 6D we show that increased sparseness, achieved by reducing the convergence onto Golgi cells from *c*_*u*_ = 100 to *c*_*u*_ = 10 (light lines) increased the quality of constructed filters as in the previous model. However, this time, increasing the granular cell population size to *Nz* = 10*k* (dotted lines) has almost no beneficial effect, which can be attributed to the bottleneck effect of the small Golgi cell population of *Nq* = 100 (compare to Fig 4C, dotted lines). Here, many granule cell responses converge onto a lower dimension of signals, which decreases the fidelity. On the contrary increasing the granule cell as well as the Golgi cell population size to *Nq* = *Nz* = 10*k* increased the filter construction performance similar to before (results not shown).

Equally, further reducing the Golgi cell population to *Nq* = 10 for the default case (*Nz* = 1*k*, *c*_*u*_ = 100) enforced the bottleneck effect and strongly decreased the construction quality of slow filters (results not shown).

The effects of synaptic-weight variability in the two-population model differed for excitatory and inhibitory weights (Fig 6E). Adding a large variability to excitatory weights *v*_*u*_ = 4 increased the goodness-of-fit (light lines) just as seen in the model before. However, adding variability to inhibitory weights *v*_*w*_ = 4 decreased the quality of constructed filters (dotted lines). This can be explained by the low number of connections between Golgi and granule cells of *c*_*w*_ = 4. Using equal convergence of *c*_*w*_ = *c*_*u*_ = 20 gave equal effects in increased filter quality with increased variability for excitatory and inhibitory weights (results not shown).

#### Putative biological features increase reservoir performance

**Fig 7:**
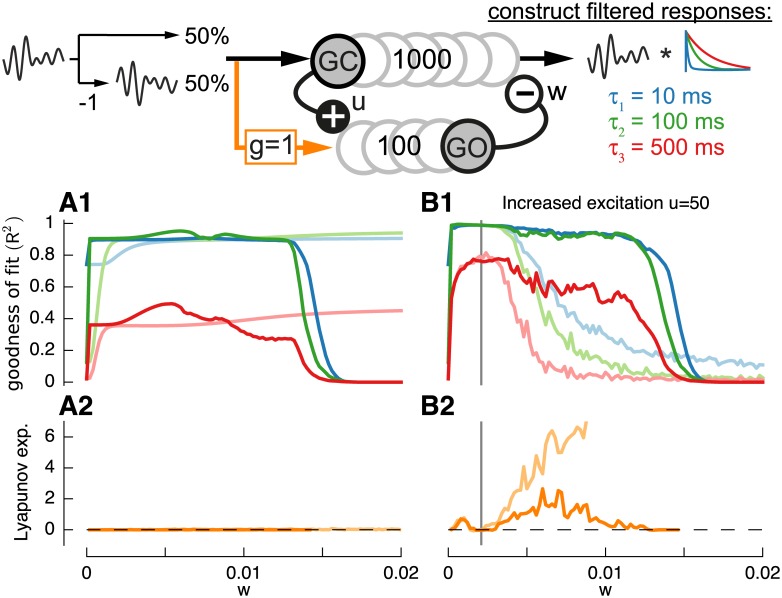
Effects of Golgi cell afferent excitation on filter construction performance. As previously, target filters used are *τ*_1_ = 10*ms* (blue), *τ*_2_ = 100*ms* (green) and *τ*_3_ = 500*ms* (red). Default synaptic time constants: *τ*_*w*_ = 50*ms*, *τ*_*u*_ = 1*ms*. **A,B**: Goodness of fit (*R* ^2^) (**A1,B1**) and Lyapunov exponents (**A2,B2**) for default network (light lines) and network with external excitation of Golgi cells (dark lines) with either default excitation of *u* = 0.1 (**A**) or increased excitation *u* = 50 (**B**).

**Fig 8:**
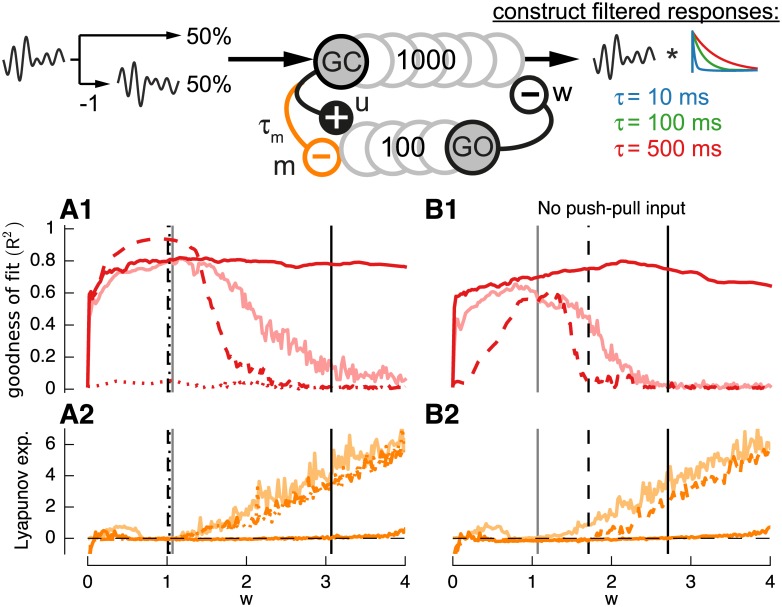
Effect of mGluR2 receptor activated GIRK channel inhibition of Golgi cells on filter construction performance. For clarity only most affected target filter *τ*_3_ = 500*ms* (red) is shown. Default synaptic time constants: *τ*_*w*_ = 50*ms*, *τ*_*u*_ = 1*ms*. **A,B**: Goodness of fit (*R* ^2^) (**A1**) and Lyapunov exponents (**A2**) for default network (light red lines) and network with mGluR2 receptor activated GIRK channel inhibition of Golgi cells (*m* = 0.003) either without (*v*_*m*_ = 0) (dotted red lines) or small (*v*_*m*_ = 0.1) (dashed red lines) weight variability. Additionally results with mGluR2 dynamics in only 50% of the Golgi cells (solid red lines) (*Pr*(m = 0) = 0.5, *v*_*m*_ = 0.1). **B** shows results for networks without push-pull input coding.

Finally, we investigate modifications to the network configurations that significantly influence filter construction (Figs 7 and 8). Default values for synaptic time constants were *τ*_*u*_ = 1*ms* and *τ*_*w*_ = 50*ms* and results are compared to a control network without modifications (light lines).

The first influential configuration is excitatory input to Golgi cells. While this property is often omitted for simplicity in granular-layer networks [20] it had a substantial impact on filter construction (Fig 7). Simply adding excitation to the Golgi cell population (*g* = 1, see Methods, equation (3) and keeping all other parameters identical prevented the system from entering into the edge-of-chaos regime (Fig 7A2) by inhibiting any activity due to the strong direct activation of Golgi cells. The only possible filter construction, which was however very poor, could be achieved during low inhibition with *w* < 0.015 (Fig 7A1, dark lines). There are several ways to increase filter construction performance during Golgi cells afferent excitation. The most obvious case being the increase of afferent Granule cell excitation, which counteracts Golgi cell inhibition and prevents Granule cells from being silenced (not shown). Another interesting possibility however is to increase recurrent Golgi cell excitation which shifts the edge-of-chaos to lower values of *w*. Increasing this weight of excitation to *u* = 50 resulted in an effective excitatory weight from granule cell to Golgi cell that was equal to the afferent Golgi cell excitation ($\frac{2}{c_{u}}u\;=\;1$, see Methods) and lead to an interesting effect on filter construction performance (Fig 7B, dark lines). While the same performance as without afferent excitation (light lines) was achieved, the decline in goodness-of-fit was postponed until larger values of *w*. The afferent excitation that effectively counteracted the generation of chaotic behavior kept the state of the network closer to the edge-of-chaos for a wider range of *w*. This can be seen in the disrupted rise in the Lyapunov exponent (Fig 7B2, dark orange lines).

A second property that also had a large influence on the filter construction was the inhibition of Golgi cells by granule cell input via mGluR2 receptor activated GIRK channels [31,32]. This property was modeled by a slow inhibitory process with *τ*_*m*_ = 50*ms* in addition to the short excitatory process (*τ*_*u*_ = 1*ms*) at the connection from granule to Golgi cell (Fig 8, top). For clarity only results for the most affected slow filter (*τ*_3_ = 500*ms*) will be shown. While initially this concurrent inhibition resulted in a strong reduction of filter construction performance (Fig 8A, dotted red lines) adding weight variability (*v*_*m*_ = 0.1) lead to a strong improvement of the overall goodness-of-fit (dashed red lines). Since some Golgi cells have been shown not to express mGluR2 [31,51] we further tested the effect of only 50% of the Golgi cells receiving mGluR2 inhibition (Fig 7A, solid red lines). While this did not massively improve filter quality compared to the baseline condition (compare to light lines) it delayed the development of chaotic behavior. The greatest beneficial effect of mGluR2 however was observed in the absence of push-pull coding (Fig 8B). Here, but only for the case of mGluR2 inhibition to half of the Golgi population, push-pull coding input is almost made redundant (solid red lines). However even with these improvements, as before, filter construction was strongly reduced with positive-LASSO regression (not shown). While the addition of variable mGluR2 inhibition did not change the qualitative behavior of single cell responses it increased the variability of damped oscillations for granule and Golgi cell responses (not shown).

#### Effect of output feedback

For some reservoir computing applications, e.g. pattern generation, the learned output signal is fed back into the reservoir leading to a recurrence between the reservoir and the trained readout [27,48,49]. In the cerebellum a corresponding connection that would allow for a similar recurrence is the nucleocortical pathway that consists of inhibitory and excitatory connections from cerebellar nucleus to the granular layer [52–54]. This connection thus supports a putative recurrent loop that sends the trained signals from Purkinje cells via cerebellar nucleus neurons back to the granular layer reservoir.

**Fig 9:**
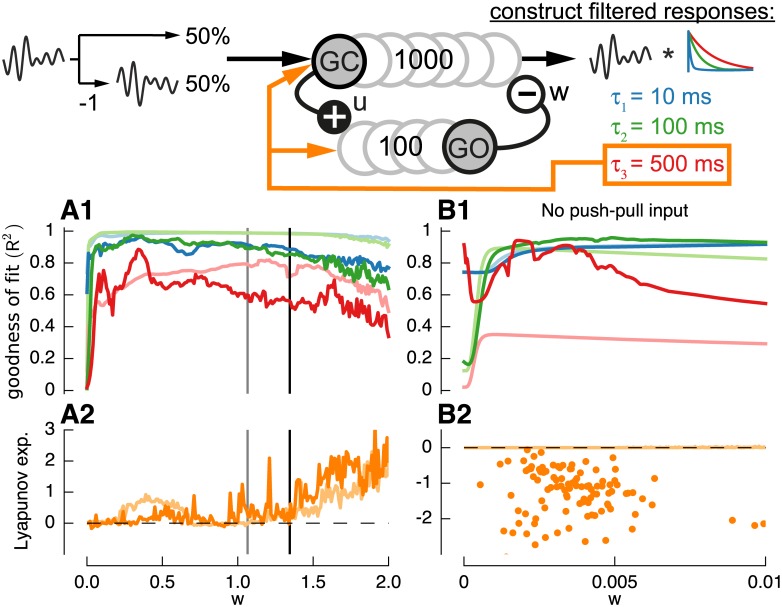
Effect of output-feedback on filter construction performance. As previously, target filters used are *τ*_1_ = 10*ms* (blue), *τ*_2_ = 100*ms* (green) and *τ*_3_ = 500*ms* (red). Default synaptic time constants: *τ*_*w*_ = 50*ms*, *τ*_*u*_ = 1*ms*. **A,B**: Goodness of fit (*R* ^2^) (**A1,B1**) and Lyapunov exponents (**A2,B2**) for default network (light lines) and network having output-feedback of the slowest filter response (*τ*_3_ = 500*ms*) to granule and Golgi cells (dark lines) with either push-pull input (**A**) or without (**B**).

While information on the exact distribution of source and target neuron properties for the nucleocortical pathway is sparse we tested the effect of output-feedback onto granule and Golgi cells using 50% excitatory and inhibitory connections (Fig 9). When using the output of the slowest filter (*τ* = 500*ms*) as feedback (Fig 9A, dark lines) the maximum performance increased for the slowest filter but slightly decreased for the two other filters (compared to case without feedback, light lines). This effect can be explained by the increase of delayed signals that are now being fed back through the slow filter output. Furthermore the maximum of filter construction quality is now well shifted away from the edge-of-chaos into more stable regimes. The preference for more stable networks with output-feedback becomes even more evident when push-pull input is removed (Fig 9B, dark lines). Here, even in the absence of any connections between Golgi and granule cells (*w* = 0) the slow filter (dark red line) can be well constructed due to the memory created by the recurrent loop between output and input, similar to a neural integrator [55]. The goodness of fit for the other two filters is however closer to the previous results (Fig 9B1, compare dark blue and green to light lines). In this regime (*w*<0.01) the reservoir with output-feedback is very stable (Fig 9B2) resulting in Lyapunov exponents of negative infinity (i.e. the Euclidian distance between perturbed and unperturbed simulation decays to 0). Interestingly however the filter performance reaches goodness-of-fit values above 80% for all three filters (Fig 9B1, dark lines) only when the Lyapunov exponents have values closer to 0 and thus the reservoir is not completely stable (Fig 9B2, dark orange dots).

## Discussion

Here we have investigated whether simplified models of the recurrent neural network (RNN) formed by the cerebellar granular layer are capable of generating signals that can be used to construct linear exponential filters with different time constants. We consider first the issues raised by our methods of simulation and analysis, then how the findings relate to previous theoretical and experimental studies, and finally the implications of the findings for future work.

### Method of simulation and analysis

#### Intermediate modeling approach

The cerebellum has been modeled at very different levels of detail, ranging from linear systems (e.g. the adaptive-filter model [9]) to compartmental neurons (e.g. Masoli et al’s recent model of the Purkinje cell [56]). A recurring problem is that while abstract models can be shown to have particular computational competencies it is generally not clear how they could be implemented biologically, whereas detailed models are more biologically realistic but their computational properties are often obscure and in any case dependent in an unknown way on a huge number of parameters.

A possible way round this problem is to use intermediate level models, intended on the one hand to be simple enough for their properties to be systematically investigated, yet on the other to have properties closer to biological reality than the abstract original. The abstract models we want to improve are those that require the granular layer to recode mossy-fibre inputs (see below) in a manner that enables Purkinje cells to learn the response appropriate for a particular task (e.g. adaptive filter model). To address this issue we chose an intermediate level model using a recurrent Artificial Neural Network (RNN) representation that is accepted as a potentially useful tool for investigating signal processing not only in the granular layer [15,17,26] but also other parts of the brain like the visual cortex [26]. The argument would be that since the properties of these RNNs are at present poorly understood, it is appropriate to explore the computational properties of relatively simple networks first. The gain in understanding is more important than the lack of biological fidelity.

We think that by incorporating more and more realistic structural properties in future versions of this model we can highlight possible differences between random connectivity and structure, a valuable insight in itself.

#### Network size

Although the largest population size of 10k neurons used here is probably rather smaller than the typical number of granule cells acting as a reservoir for a particular cerebellar microzone we have shown that it is nevertheless capable of implementing reservoirs from which the filters necessary to implement cerebellar functions can be constructed. This is compatible with the cerebellar ‘chip’ metaphor [56] where microzones are indicated to implement separate functions [4]. By exploring different network sizes and lower convergence ratios (Figs 4C and 6D) we further indicate that the described reservoir effect does not diminish but is rather improved by up-scaling. This extra capacity may give the reservoir capabilities for implementing a wider range of basis functions, such as non-linear or multimodal bases. However, one has to be cautious when comparing the number of simulated rate neurons and effective individual biological spiking neurons. While rate neurons are a simplification that offer perfect signal transmission, this is not the case with individual spiking neurons like granule cells where one rate neuron unit would have to be constituted by several biological neurons to achieve the same signal transmission fidelity [38].

Our 1k and 10k models do not differ much from the population size in spiking models of the cerebellum with 4k [50] and 10k [11,13,14]. Even scaling up by a factor of 100 from 10k to 1 Million cells, allowing for more realistic divergence ratios, only gave modest increase in performance for eye-blink conditioning [57], further suggesting that the basic principles of cerebellar computation can already be captured in rather small networks.

#### Analysis with LASSO-regression

While actual synapses are constrained to be either excitatory or inhibitory, regression methods in general, and LASSO-regression in particular, use positive and negative weights to construct the desired output signal from the input signals. This assumption can however be justified if there is an appropriate inhibitory pathway between granule and Purkinje cells, now shown to be provided via molecular layer interneurons [42,44] which have been indicated to be essential for cerebellar motor learning [58]. Furthermore we showed that when using push-pull coding, filters could be constructed using LASSO-regression even with positive coefficients, i.e. excitatory synapses, only.

This study tries not to claim that LASSO-regression is a substitute function for learning at Purkinje cells and it doesn’t try to answer how plasticity at Purkinje cells is actually capable to construct the filters from the given reservoir. This is even more the case during the simulation with output-feedback where learning is very unstable and teacher forcing was applied. The given method is merely a powerful and descriptive way to analyze the performance of the granular layer RNN and shows whether it would be able to provide the necessary components of computation and memory.

In the adaptive filter the learning mechanism at Purkinje cell synapses is modeled using the covariance learning rule which is compatible with known properties of LTP and LTD at PF/PC and PF/MLI synapses. With this assumption it can be shown that the end state after learning is least squares optimal [59] and that the unavoidable presence of noise on parallel fiber inputs drives synaptic weights to the smallest values compatible with task accuracy [60]. For efficiency in dealing with large populations, and to avoid overfitting, this learning process has been modeled using LASSO regression. This is a least-squares minimization procedure with a regularization term to keep reconstruction weights small and hence has essentially the same end state as covariance learning in the presence of noise. Since regularization tends to reduce fitting accuracy we believe this is a conservative method for quantifying reservoir performance.

### Difference between one- and two-population models

The one-population model demonstrated the properties of a homogeneous reservoir dominated by recurrent inhibition. However in the cerebellum recurrent inhibition is implemented as a relay via a second population of Golgi cells. To test the effect of this layered recurrence we considered a two-population model and found that reservoir behavior was generally preserved. We did observe three differences between the two types of networks: 1. A slow synaptic time constant for Golgi cell excitation decreases the quality of fast filter construction (Fig 6C). 2. The lower number of Golgi cells can create a bottleneck in filter fidelity when few Golgi cells sample input from a large number of granule cells (Fig 4C). 3. Direct afferent Golgi cell excitation can decrease chaotic behavior and is beneficial for filter construction by prolonging the edge-of-chaos regime. These issues are further discussed below.

### Theoretical findings

Early models of the cerebellar microcircuit focused on its ability to adaptively process static patterns. Subsequently Fujita [3] described a cerebellar model that could adaptively process time-varying analogue signals, based on the adaptive linear filter used in signal processing and control engineering [61]. In Fujita’s model the granular layer recurrent neuronal network (RNN) generated a set of parallel-fiber outputs for a given mossy-fiber input which by linear summation at the Purkinje cell allowed the microcircuit to adaptively transform time-varying inputs into the specific time-varying outputs required for any given signal-processing task.

Fujita’s adaptive-filter model required the granular layer to generate computationally adequate sets of parallel-fiber outputs in a biologically realistic way [4]. Subsequent extensions of his work initially focused on computational adequacy, suggesting suitable basis functions for transforming mossy-fiber inputs that included tapped delay lines, spectral timing, and sinusoids (references in [8–10]). However, it was not usually made clear how these functions could be generated by a plausible neuronal network, raising concerns that the approach was unrealistic [11] and excessively ‘top-down’ [14]. The preferred alternative was ‘bottom-up’ modeling in which temporal-processing properties emerged from biologically detailed models of the granular-layer RNN rather than being imposed by a priori theoretical considerations [13,14,62]. These models successfully learned an eyeblink-conditioning task by generating a suitably delayed output timed to coincide with the arrival of the unconditioned stimulus.

Yamazaki and Tanaka [15] investigated the generic time-coding properties of simplified RNNs, with (usually) 1000 rate-coding neurons receiving excitatory afferent inputs and recurrent inhibitory inputs from other neurons (cf. first model here). These RNNs could generate a sequence of activity patterns that never recurred, a sequence that could be triggered reliably by a strong transient input signal. Such networks could therefore be used to encode the passage of time for any task that required it. Related results were found for RNNs of integrate-and-fire model neurons [16], and for a more realistic two-layer RNN embedded in a spiking model of the entire cerebellar microcircuit [63].

#### Reservoir computing

Yamazaki and Tanaka [17] noted the resemblance between their approach and that of reservoir computing [28,64–66], where artificial RNNs consisting of either non-spiking nodes (echo state networks) or spiking model neurons (liquid state machines) are used to generate a rich high dimensional “reservoir” of dynamic responses to a temporally varying input. These responses are weighted and linearly summed by a plastic readout unit, and the weights adjusted so that the system’s output is as close as possible to a desired output. Suitable RNNs exhibit temporal memory, in that short-duration inputs can generate long-duration outputs, and can be used to model nonlinear functions and thus act as a non-linear adaptive filter [25,49,66,67]. These capabilities have been applied to a range of problems such as robot motor control that are thought to involve the cerebellum in their biological equivalents [21]. Yamazaki and Tanaka [17] applied the generic reservoir-computing framework specifically to the cerebellum, and showed that a cerebellar-inspired RNN was capable to learn and represent Boolean functions.

However, neither this study nor its predecessors addressed the generic issue of temporal processing (see Introduction). In the present study we therefore showed that cerebellar-inspired RNNs could in fact generate a range of temporally-varying outputs, in this case exponentials with different time constants. Exponentials are suitable basis functions for linear transformations and have been shown to be effective in a linear model of the cerebellum for adaptation of the vestibulo-ocular reflex [9]. Here we show how these ‘top-down’ functions can be generated by RNNs resembling previous ‘bottom-up’ models, thus starting to bridge the gap between the two approaches outlined above.

We also found that the ability to generate exponential functions depended on the mean amount of feedback in the network. Theoretical investigations of RNNs, both from within the reservoir-computing framework [23–25] and outside it [26,29,30,68,69] suggest that, in very broad terms, the outputs of networks with weak recurrent connections tend to reproduce the temporal structure of their input, whereas when the recurrent connections are strong the outputs are determined primarily by the network’s internal structure. Between these two extremes, a suitably connected RNN may be able to reproducibly deliver a rich variety of temporal responses to an input stimulus. In the present study we found that it was RNNs with small, negative values of Lyapunov exponents, representing this intermediate ‘edge-of-chaos’ region that generated long-lasting and complex responses to transient inputs. In contrast, Yamazaki and Tanaka’s ‘passage-of-time’ RNNs appear to operate where the network’s internal state dominates, conveying little information about the temporal structure of its inputs. Yamazaki and Tanaka [15] have in fact argued that this chaotic network state is the preferred network state to implement an internal clock, but our results show that it is disadvantageous when a filter applied to a continuous signal has to be implemented.

Previous work has already highlighted the advantages of reservoir networks for various computational tasks [23–28] and established the usefulness of different stable, critical or chaotic regimes [22,29,30] generated by changing the weights inside the reservoir. This however was almost exclusively done for generic networks where neurons are always connected by negative and positive weights [27] or include both mutual exciting and inhibiting neurons in the randomly connected reservoir networks [28]. Our work is the first to thoroughly examine the performance in network regimes while accounting for the restrictions of basic cerebellar connectivity where reservoir granule cells do only inhibit through feedback of interneurons (Golgi cells), but do not excite each other. This effectively leads to a reservoir network of inhibitory interneurons (Figs 2, 3, 4 and 5). By incorporating Golgi cells in the model (Figs 6, 7, 8 and 9) we further showed the influence of inhibitory neuron properties like population size, direct excitation, synaptic time constant and mGluR2 inhibition on reservoir performance and stability.

### Experimental findings

The main theoretical conclusion is that with appropriate network parameters the granular-layer RNN can in principle generate the outputs needed for an adaptive filter. It also indicates what those outputs might look like. We now consider experimental evidence bearing on these two points. For convenience, evidence from detailed models of the granular-layer that are concerned primarily with its electrophysiology (as opposed to the functional models discussed above) is also included in this experimental section.

#### Direct evidence: Recordings from granular layer

In contrast to previous ‘top-down’ models where individual granule cells code specific basis functions [8], here basis functions are carried by populations of granule cells and the firing patterns of individual cells (Fig 2B) are hard to interpret. But a prediction for experimental testing can still be made, which is that the same mossy fiber input should produce a variety of granule-cell responses, some of which outlast the input.

The responses to forelimb stimulation of granule cells in the C3 zone of decerebrate cats are very homogeneous, apparently reflecting only a slight transformation of their mossy-fiber inputs [70–72]. Similar high-fidelity transmission has also been observed for granule cells in crus I and IIa of anaesthetized rats that respond to perioral or vibrissal stimulation [73]. In contrast, Holtzman et al. [32] found heterogeneous responses of granule cells in crus Ic/IIa/b of anesthetized rats to brief hindlimb stimulation. In particular, they show various combinations of short-, long lasting and early-, late excitation and depression, which is very compatible with our results for simulations with feedback inhibition (Fig 2B). Moreover, it appeared that long-lasting granule-cell responses were related to long-lasting depression of Golgi-cell firing, which in turn was produced by glutamate released from granule cells acting via mGluR2 receptors. Heterogeneous firing patterns have also been described for granule cells in the flocculus of awake rabbits in response to changes in head velocity [74]. However, it is not yet clear how much of this heterogeneity comes from diversity of mossy-fiber input or the action of unipolar brush cells rather than feedback from Golgi cells (further discussion below and [38]).

#### Indirect evidence: General connectivity

In contrast to common reservoir-computing networks, in the cerebellar granular layer only recurrent inhibition onto granule cells is available, posing an additional constraint for successful filter synthesis. One way of compensating for the lack of mutual excitation is employing “push-pull” input coding, in which half of the cells receive signals in phase with the input, and the other half signals that are out of phase, i.e. multiplied by –1. Direct evidence that push-pull coding is available at least in the vestibular system comes from recordings where approximately half of the mossy fibers increase firing when the head is moved ipsilateral to the recording site (type 1) and half for contralateral movement (type 2). This has been shown in recordings from the cells of origin of floccular mossy fibers in awake primate [75] and cat [76]. Furthermore also most granule cells can be separated into type 1 and type 2 activity as shown by extracellular [77] and internal EPSP recordings in anesthetized mice [78].

A recent experiment showed that rerouting of climbing fibers can switch the simple spike activity of Purkinje cells from ipsi- to contralateral [79] while maintaining reciprocal complex spike activity [80]. This change occurs without any modification of mossy fiber input, further supporting the idea that the granular layer exhibits a diverse signal reservoir that in addition can be actively shaped with plasticity controlled by climbing fiber activity.

Two other of our results on optimal Golgi cell excitation are directly supported by recent findings. First of all, optimal filter construction requires sparse connectivity arguing for local connectivity, i.e. small world connectivity and against a congregation of global activity. In support of this is the finding that Golgi cells do not only receive distal excitatory inputs from other modules via the parallel fiber pathway but more than half of its excitatory inputs are local inputs from neurons within the module via ascending axons with a connection probability of 10% [81].

Finally, the filters in our study can be constructed only using a fraction of the provided granule cell signals which is compatible with the high proportion of silent synapses found between parallel fibers and Purkinje cells [42–45].

#### Indirect evidence: Synaptic properties

A necessity for our proposed model is the presence of a strong excitatory—inhibitory loop between granule and Golgi cells. Direct evidence for this comes from recent experiments [81] showing that during sustained mossy fiber activity half of the excitatory charge input to Golgi cells comes from granule cell synapses. Furthermore, more than half of the these local connections are from local ascending axon connections on basolateral Golgi cells dendrites with decay time constants faster than 1.2 ms supporting our assumption of fast excitation.

One property that naturally increases the range of expansion recoding i.e. the ability to construct long filters is to increase the time constant of the synaptic inhibition. GABAergic inhibition of granule cells is usually reported to be strong enough to dynamically regulate output of granule cells [82] and to have a large slow component that is manly governed by the spillover activation of *α*_6_GABA_*A*_ receptors [83].

We show that compensation for the lack of mutual excitation can also be achieved by paradoxical inhibition of Golgi cells due to mGluR2 receptor activated GIRK channels [31,32]. In the presence of push-pull input mGluR2 activation can improve filter construction, but only when the density of mGluR2 and/or GIRK channels is variable across Golgi cells. Further increasing the variability of mGluR2 to the point where only 50% of Golgi cells receive inhibitory input can even compensate for the absence of push-pull coding by bringing the connectivity closer to that of traditional reservoir models where connections are either excitatory or inhibitory. This strong variability across cells can be justified by the finding that not all Golgi cells express mGluR2 [31,51].

Very compatible with our results are the findings of long-lasting alternating excitation and inhibition to brief sensory stimulation due to mutual inhibition of Golgi cells by mGluR2 and granule cells by GABA [32]. However since the latter study based their identification of Golgi cells on the occurrence of long lasting depression due to putative mGluR2 inhibition, recordings of Golgi cells without mGluR2 expression, which we find to be very important for filter construction, are not available.

#### Indirect evidence: Output feedback

A further property that can greatly improve filter construction is output-feedback via the nucleocortical pathway sending trained signals from Purkinje cells via cerebellar nucleus neurons back to the granular layer reservoir [52–54]. We report that this recurrent loop could increase reservoir memory especially in the absence of push-pull input where it does require a more stable network with lower inhibitory gain. However, learning under this condition is difficult. While teacher forcing, a strategy used in reservoir computing is able to tackle unstable learning tasks [27] by providing the target output signals before the weights are even learned, it remains to be shown if learning at Purkinje synapses itself can be stable during output feedback.

### Implications of findings

In all networks and configurations considered the single cell activity of granule and Golgi cells at the edge-of-chaos showed complex but stable, long lasting damped oscillations that were the effect of inhibition and dis-inhibition (only shown for the one-population model, Fig 2B). The similarity between Golgi and granule cell responses is easily graspable when one considers that for the default two-population network Golgi cells merely relay signals from granule cells und thus have to show similar activity. On the other hand for Golgi cells with added mGluR2 dynamics they themselves become prone to inhibition and dis-inhibition. This study thus suggests that one indicator for the presence of reservoir computation in a certain area of the cerebellum would be the similarity in heterogeneity and timing of the responses for granule and Golgi cells.

This comparison however must not be made based on the spike activity but on the modulated signals. One explanation for the often-reported bursting responses in granule cells compared to Golgi cells could be the lower baseline/background activity of the former cells [77]. While the signal is carried and hidden by the higher spike rate in Golgi cells, granule cells would ultimately only spike during phases of strong dis-inhibition, which would effectively resemble bursting behavior. This would be even further increased if the operation point of the network is not close to the edge-of-chaos but in the chaotic regime. A further evaluation of these properties will however require the inclusion of spiking neurons in future studies.

While the present study only focuses on the interaction between granule and Golgi cells the inclusion of other identified neurons might improve filter construction properties. Glycinergic Lugaro cells which have been found to increase the long-lasting depression of Golgi cells [32] and various other non-traditional interneurons like globular [84] or perivascular neurons [85] might further improve the reservoir performance by contributing to the inhibitory circuit. Furthermore, in some areas of cerebellar cortex, particularly in the vestibulo-cerebellum (e.g. [86]), a substantial proportion of mossy-fiber input is processed and relayed by unipolar brush cells (UBC) which are thought to prolong and diversify the input signals [87]. In addition a recently discovered timing mechanism intrinsic to Purkinje cells [88] would potentially further increase the heterogeneity of the granular layer reservoir signals.

Although the edge-of-chaos criterion is not a universal predictor of maximal computational performance [22] we find that it applies for most of the configurations considered here. With this requirement however the question arises how the granular layer network can be adjusted to operate in this computationally powerful regime. While the easiest way to achieve this is to change properties inside the loop like weights at Golgi cell—granule cell synapses or the convergence ratio of Golgi cell excitation we show that also external mechanisms like noise, input variability and especially mossy fiber—Golgi cell exaction can shift the network state. The question however remains how and if the cerebellar granule layer network can be automatically tuned to operate close to the edge-of-chaos. Possible mechanism that could help achieve this are synaptic long or short-term plasticity.
